# Effects of steroids on reintubation and post-extubation stridor in adults: meta-analysis of randomised controlled trials

**DOI:** 10.1186/cc7772

**Published:** 2009-04-03

**Authors:** Samir Jaber, Boris Jung, Gérald Chanques, Francis Bonnet, Emmanuel Marret

**Affiliations:** 1 Department of Anaesthesiology and Critical Care, University Saint Eloi Hospital, 80, avenue Augustin Fliche; University of Montpellier I; 34295 Montpellier Cedex 5, France; 2 Department of Anesthesiology and Critical Care, Tenon University Hospital, Assistance Publique Hôpitaux de Paris, and INSERM U 707 Université Pierre et Marie Curie, Paris 6, Paris, France

## Abstract

**Introduction:**

The efficacy of steroid administration before planned tracheal extubation in critical care patients remains controversial with respect to the selection of patients most likely to benefit from this treatment.

**Methods:**

We performed an extensive literature search for adult trials testing steroids versus placebo to prevent reintubation or laryngeal dyspnoea. Studies were evaluated on a five-point scale based on randomisation, double-blinding and follow-up. Our analysis included trials having a score three or higher with patients mechanically ventilated for at least 24 hours and treated with steroids before extubation, taking into account the time of their administration (early vs late) and if the population selected was at risk or not.

**Results:**

Seven prospective, randomised, double-blinded trials, including 1846 patients, (949 of which received steroids) were selected. Overall, steroids significantly decreased the risk of reintubation (relative risk (RR) = 0.58, 95% confidence interval (CI) = 0.41 to 0.81; number-needed-to-treat (NNT) = 28, 95% CI = 20 to 61) and stridor (RR = 0.48, 95% CI = 0.26 to 0.87; NNT = 11, 95% CI = 8 to 42). The effect of steroids on reintubation and stridor was more pronounced for selected high-risk patients, as determined by a reduced cuff leak volume (RR = 0.38, 95% CI = 0.21 to 0.72; NNT = 9, 95% CI = 7 to 19; and RR = 0.40, 95% CI = 0.25 to 0.63; NNT = 5, 95% CI = 4 to 8, respectively). In contrast, steroid benefit was unclear when trials did not select patients for their risk of reintubation (RR = 0.67, 95% CI = 0.45 to 1.00; NNT = 44, 95% CI ≥ 26 to infinity) or stridor (RR = 0.56, 95% CI = 0.20 to 1.55).

**Conclusions:**

The efficacy of steroids to prevent stridor and reintubation was only observed in a high-risk population, as identified by the cuff-leak test and when it was administered at least four hours before extubation. The benefit of steroids remains unclear when patients at high risk are not selected.

## Introduction

Post-extubation stridor associated with post-extubation laryngeal oedema is one of the most frequent causes of reintubation in the intensive care unit (ICU) [1-7]. Reintubation may result in increased morbidity (for example, nosocomial infection, prolonged length of ICU stay, additional costs) and mortality [1-4,6,7]. The prevalence of post-extubation stridor ranges between 6 and 37% of intubated ICU patients [5,8-13], depending on the studied population (those at high risk or not). Controversy still exists about the effectiveness of prophylactic steroid therapy to prevent occurrence of both post-extubation stridor and related reintubation in both patients selected because they are at high risk of stridor and reintubation [8,9,13] and non-selected patients [10-12,14].

Two recent meta-analyses [15,16], based on original papers published up to 2007, have been performed. They report contradictory conclusions regarding the efficacy and safety of prophylactic steroid therapy in preventing post-extubation laryngeal oedema and the need for reintubation in adult ICU patients. Fan and colleagues [15] have suggested, regarding the most recent clinical trials, that prophylactic steroid therapy can reduce the incidence of post-extubation laryngeal oedema and the subsequent need for reintubation in mechanically ventilated patients. In contrast, Markovitz and colleagues [16] concluded that using steroids to prevent (or treat) stridor after extubation has not proven effective for neonates, children or adults. By reporting conflicting results, recent trials [8,9,13] and the two meta-analyses [15,16] have intensified the debate surrounding the use of prophylactic steroid therapy to prevent both post-extubation stridor occurrence and reintubation. Moreover, the meta-analyses results were pooled from trials which included selection of patients at risk of post-extubation stridor development [8,9,13] and unselected patients with an no risk of post-extubation stridor development [10-12,14] and allowed for very different steroid administration regimens (well in advance of extubation or immediately before). Indeed, the anti-inflammatory effect of steroids, the main mechanism responsible for reduction of post-extubation laryngeal oedema, is time-course dependant [17,18]. Although the two meta-analyses [15,16] allowed for these differences, they did not perform subgroup analyses of the early versus late steroid administration nor for selected high-risk patients versus unselected patients. Finally in 2007, two additional randomised clinical trials (RCTs) were presented in abstract form but were not included in these two meta-analyses [8,14]. Thus, we performed a quantitative meta-analysis to evaluate the effectiveness of prophylactic steroid therapy to prevent reintubation and post-extubation stridor, taking into account the studied populations (at risk to develop post-extubation stridor or not) and the steroid administration regimen (pre-extubation early versus late).

## Materials and methods

QUOROM standards were followed during all phases of the design and implementation of this meta-analysis [19].

### Identification of the studies

Three electronic databases were searched via the Internet for studies published between January 1966 and November 2008: PubMed^® ^(MEDLINE/*Index Medicus*), the Cochrane Controlled Trials Register published by the Cochrane Library and EMBASE. The Medical Subject Heading terms used for the search were *steroids *and *extubation, adults and randomized controlled trials*. Supplementary manuscripts were searched by changing the Medical Subject Heading term *steroids *to *dexamethasone*, *prednisolone*, *methylprednisolone *or *hydrocortisone*. Additional references were retrieved by clicking on the 'related articles' hyperlinks in Medline and by manually searching reference lists in original published articles, review articles and correspondence. To complete the search with the inclusion of non-published trials, abstracts presented at different critical care meetings (American Thoracic Society, Society of Critical Care Medicine, American Society of Anesthesiology, European Society of Anaesthesiology, European Society of Intensive Care Medicine, International Symposium on Intensive Care and Emergency Medicine, Societé Française d'Anesthésie-Réanimation and Société de Réanimation en Langue Française) were also screened. For abstracts, only the past three years were consulted. For some trials, the authors were contacted for additional information on the results [8,14].

### Quality assessment of the studies

Each study was subjected to quality assessment by two investigators (SJ and BJ) who were not blinded to the authors or results. Disagreements between the two investigators were resolved by discussion. In the case of persistent disagreement, a third reviewer (EM) helped to reach a consensus after separately reviewing the report. Each article was scored using a five-point scale that evaluates randomisation, blinding and completeness of patient follow-up (Jadad scale) [20]. One point was given if the study was described as randomised. An additional point was given if the randomisation method was described and was appropriate (for example, computer-generated table of random numbers), whereas a point was subtracted if the randomisation method was described and inappropriate (for example, alternate allocation or allocation by date of birth). Similarly, one point was assigned to studies described as double-blinded, two points were assigned to studies for which the double-blinding method was described and appropriate (for example, identical placebo, active placebo, double-dummy) and zero points were assigned to studies for which the double-blinding method was described and inappropriate. One point was given if the article specified the numbers of and reasons for withdrawals and dropouts. Thus, the minimum score for a randomised study was one and the highest possible score was five. We included studies with a score of three or greater [20].

### Selection criteria

Criteria for study selection were as follows: randomised, double-blind design; quality assessment score of three or greater [20]; duration of mechanical ventilation longer than 24 hours; steroids administrated before a planned extubation.

Criteria for study exclusion were a score of two or less on the three-item Jadad quality five-point scale; duration of mechanical ventilation less than 24 hours (for example, mechanical ventilation for anaesthesia); trials that studied steroid administration for the prevention of pulmonary fibrosis (for example, excessive fibroproliferation or bronchopulmonary dysplasia); paediatric or neonatal patients.

### Outcome measures

The primary evaluation criterion was the incidence of reintubation. The other endpoints of post-extubation stridor, duration of ICU stay and mortality were analysed. When trials compared more than two groups, data were extracted into two groups: steroid and control. In dose-ranging studies with a placebo group, we extracted the events of the control group and pooled the steroid groups. When authors compared two types of administration with the same dose of steroids (single injection vs. intermittent or bolus group), patients receiving steroids were pooled and compared with those receiving placebo.

Sensitivity analysis was performed to explore the effect of steroid in different populations, namely in trials which selected patients at high risk for reintubation or not. Similarly, subgroup analysis for time of administration was conducted in groups of patients who received steroids 'late' (less than two hours before extubation) or 'early' (more than four hours before extubation).

### Statistics

Data were extracted as they were reported in the original paper or based on the answers of the authors to our queries. The Mantel-Haenszel-like procedure for relative risk (RR) was used to pool RRs [21]. Analyses were performed with Rev Man review manager (version 4.2, Cochrane Collaboration, The Nordic Cochrane Centre, Copenhagen). The RRs (and 95% confidence intervals (CI)) were calculated, and the results were expressed graphically. All criteria were analysed separately. A random-effects analysis was conducted if the result of a Q Cochrane heterogeneity test was significant (*P *< 0.1) and heterogeneity was quantified by I^2 ^[22]. For the significant criteria, we computed the number needed to treat (NNT) as the inverse of the difference of the proportion of patients who had any event in the steroid groups and the control groups. CIs of the NNT were constructed by inverting and exchanging the limits of the 95% CI for the RR. The NNT and 95% CI were calculated with the Internet-based program Visual Rx [23]. All tests were two sided, and *P *values less than 0.05 were considered statistically significant.

A funnel plot (plot of treatment effect against trial precision) was also created to determine the presence of publication bias and other possible biases (English language, citation and multiple publication), true heterogeneity, data irregularities and choice of effect measure in the meta-analysis [24]. In the presence of bias that usually leads to an overestimate of the treatment effect, the funnel plot is skewed and asymmetrical. The degree of asymmetry was measured by the Egger test [25] using WeasyMA software (ClinInfo, Lyon, France) [26]. A *P *value less than 0.1 was considered statistically significant for asymmetry.

## Results

### Identification of the trials

**Figure 1:**
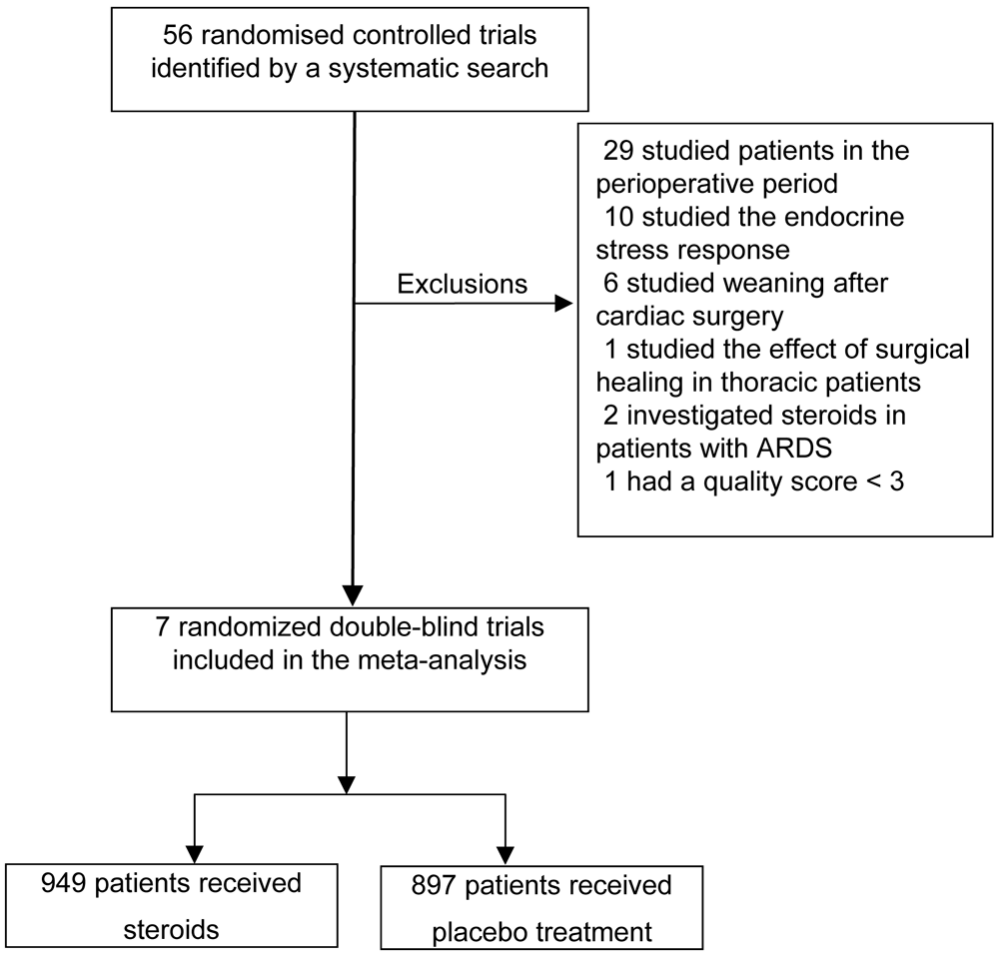
Flowchart of randomised controlled trials selected for the meta-analysis. ARDS = acute respiratory distress syndrome.

Fifty-six relevant RCTs were identified by Medline, the Cochrane Library, Embase and hand-searching. Forty-eight were excluded for the following reasons: 29 were surgical patients (evaluation of steroid neuromuscular block or steroids to prevent postoperative nausea or vomiting); 10 studies investigated the endocrine stress response; six trials evaluated the effect of steroids on ventilation weaning after cardiac surgery; two trials investigated long-term administration of steroids in patients with acute respiratory distress syndrome; and one trial studied the effect of steroids on healing after thoracic surgery (Figure 1). One RCT was excluded because the quality assessment score was less than three [26]. Two trials were found after consulting conference abstracts [8,14]. Seven studies were finally selected including 1846 adult patients. Nine hundred and forty-nine patients were included in the steroid group, versus 897 in the placebo group (Figure 1).

### Study designs and patients

The characteristics of the seven RCTs are summarised in Table 1. All seven randomised double-blinded studies were published in or after 1992. Two trials [8,14] were presented at the American Thoracic Society conference in 2007 and one author answered our queries concerning additional data [8]. The median quality score of data reporting was five (range = three to five). All studies were double-blinded; the procedure of randomisation was adequately described in five out of seven studies. Type of corticosteroid, doses, timing and duration of administration varied from one trial to another (Table 1). Three trials only included patients at high risk of distress after planned extubation based on a reduced cuff-leak volume [8,9,13]. One trial [9] had three arms; it compared patients that received a placebo with patients that received one injection of methylprednisolone (low-dose steroid arm) and patients that received four injections of corticosteroid (high-dose steroid arm); these two steroid arms were thus combined for the analyses.

**Table 1:** Characteristics of the seven adult studies included in the meta-analysis

**Author, year**	**Jadad** **scale**	**Overall** **sample** **size analysed** **(n)**	**ICU population** **and inclusion criteria**	**Duration of ventilation** **(days)** **(steroid vs placebo)**	**Steroid dose and** **regimen administration**	**Overall** **equivalent dose of** **hydrocortisone (mg)**
**Cheng and colleagues 2007 **[8]	3	71	Medical and surgical MV for more than 24 hours High risk of stridor (CLV < 24%)	NR	Methylprednisolone IV 40 mg, 4 hours before extubation	200
**Cheng and colleagues 2006 **[9]	5	128	Medical and surgical MV for more than 24 hours High risk of stridor (CLV < 24%)	7.3 ± 3.9 (1 inj) 6.3 ± 3.8 (4 inj) vs 7.1 ± 4.1 (placebo)	Methylprednisolone IV 40 mg/6 hours × 4 vs Methylprednisolone IV 40 mg – 1 injection vs placebo Started 24 hours before extubation	800 or 200
**Darmon and colleagues 1992 **[10]	5	694	Medical and surgical MV for more than 36 hours Not selected at high-risk	9.6 ± 9.7 vs 10.3 ± 10.9	Dexamethasone IV 8 mg one hour before extubation	213
**Francois and colleagues 2007 **[11]	5	698	Medical, surgical and trauma MV for more than 36 hours Not selected at high-risk	Duration of MV < 7 days: 51 vs 49% Duration of MV > 7 days: 49 vs 51%	Methylprednisolone IV 20 mg/4 hours starting 12 hours before planned extubation (last dose just before extubation)	400
**Ho and colleagues 1996 **[12]	5	77	Medical and surgical Not selected at high-risk	6.1 ± 3.8 vs 4.6 ± 4.7	Hydrocortisone IV 100 mg one hour before extubation	100
**Lee and colleagues 2007 **[13]	5	86	Medical MV for more than 48 hours High risk of stridor (CLV < 110 ml)	7.0 ± 2.0 vs 6.6 ± 2.0	Dexamethasone IV 5 mg/6 hours × 4 – started 24 hours before extubation, last dose just before extubation	533
**Shih and colleagues 2007 **[14]	3	98	Medical and surgical MV for more than 24 hours	Between 10 and 15	Hydrocortisone IV 4 injections/6 hours Started 24 hours before	NR

CLV = cuff-leak volume; ICU = intensive care unit; IV = intra-venous; MV = mechanical ventilation; NR = not reported.

Post-extubation stridor was mainly defined by the occurrence of stridor after extubation, except in two trials where the authors included patients with stridor and laryngeal obstruction dyspnoea defined by the occurrence of signs of upper airway obstruction, that is, a prolonged inspiratory phase associated with recruitment of accessory respiratory muscles [10,12]. Post-extubation laryngeal oedema was confirmed by examination using bronchoscopy or laryngoscopy in two trials [9,11].

### Outcomes

#### Outcomes according to populations included in the trials: overall, unselected and selected patients at high risk of developing post-extubation stridor and reintubation as defined by a reduced cuff-leak volume

**Figure 2:**
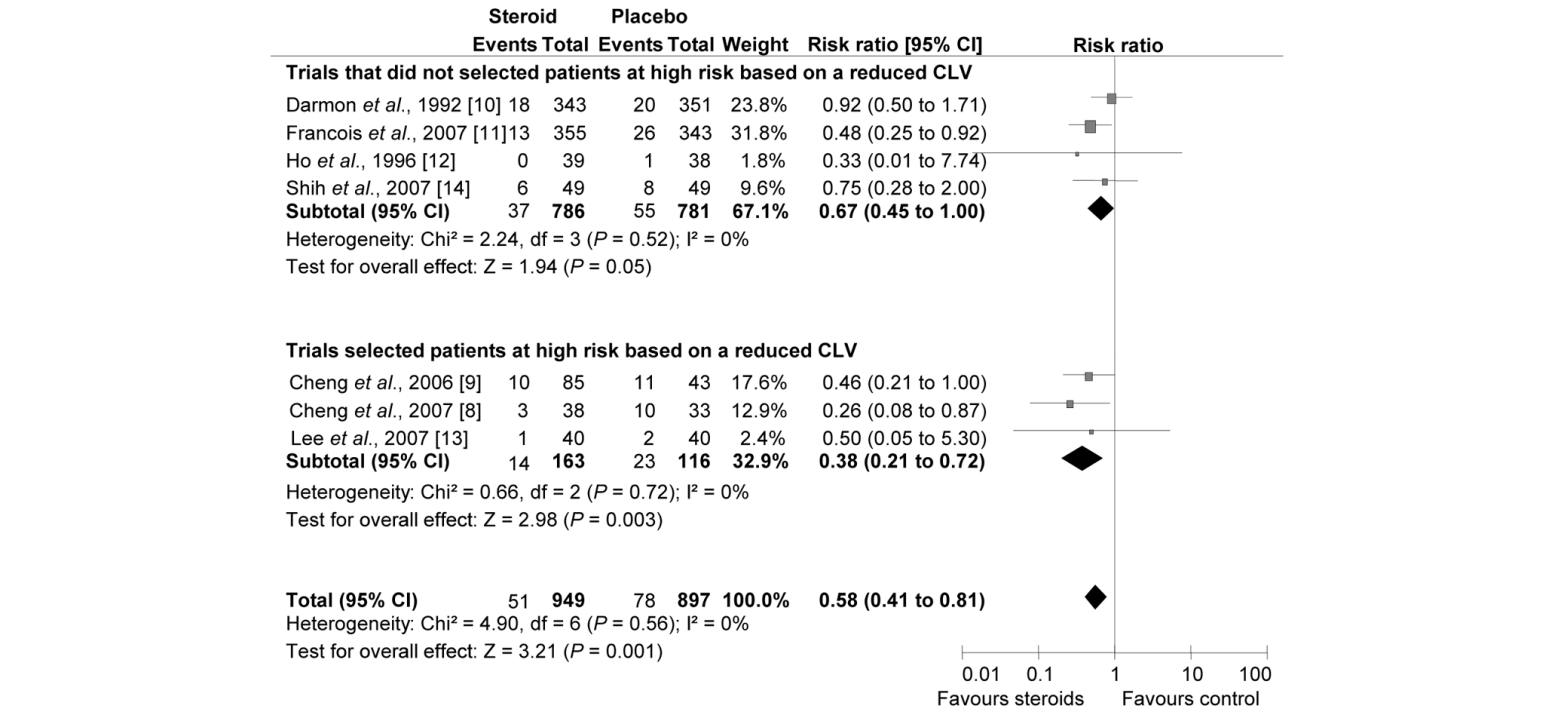
Risk of reintubation according to the studied population. Risk ratio of reintubation rate for the individual randomised controlled trials comparing steroids with control groups. Vertical line = 'no difference' point between the two groups; squares = risk ratios (the size of each square denotes the proportion of information given by each trial); diamonds = pooled risk ratios for randomised controlled trials that did not select patients at high risk (upper) and trials that did select patients at high risk, based on a reduced cuff-leak volume (CLV; lower); horizontal lines = 95% confidence intervals (CI).

The rates of reintubation were obtained for all selected trials. Figure 2 demonstrates a significant difference in the reintubation rate after a planned extubation, with 8.7% (range = 2.6% to 30.3%) in the controls and 5.4% (range = 0% to 12.9%) in the steroid-treated patients (RR = 0.58, 95% CI = 0.41 to 0.81, *P *= 0.001). This indicates a 42% decrease in the risk of reintubation. The NNT overall patients (unselected and selected patients) was 28 (95% CI = 20 to 61; Table 2). Subgroup analysis was performed by pooling trials that selected high-risk patients by measuring the leak around the deflated endotracheal tube cuff. The risk of reintubation was more greatly reduced by steroids when only trials with these high-risk patients were considered. The rate of reintubation decreased from 19.8% to 8.6% (RR = 0.38, 95% CI = 0.21 to 0.72, *P *= 0.003) The NNT of high-risk patients was 9 (95% CI = 7 to 19; Figure 2 and Table 2). In comparison, the risk reduction appears less well defined when trials did not select patients for risk of reintubation (RR = 0.67, 95% CI = 0.45 to 1.00, *P *= 0.05; NNT = 44, 95% CI ≥ 26 to infinity; Table 2).

**Table 2:** Number needed to treat with steroids to reduce reintubation and stridor in unselected, selected and overall populations

	**Unselected**	**Selected**	**Overall** **(unselected+selected)**
NNT to prevent one reintubation episode	44 (95% CI ≥ 26 to ∞)	9 (95% CI = 7 to 19)	28 (95% CI = 20 to 61)
NNT to prevent one stridor episode	Not calculated	5 (95% CI = 4 to 8)	11 (95% CI = 8 to 42)

Selected population is defined as patients at high risk of developing post-extubation stridor and reintubation in which the cuff-leak test showed absence or a low level of leak (less than 110 to 140 ml in absolute value or less than 12% to 25% in relative value).

Unselected population is defined as patients included in trials that did not use the cuff-leak test to select patients.

Overall population is defined as patients included in both trials that did use and did not use the cuff-leak test to selected patients (unselected+selected).

The NNT was calculated only when a significant result was observed.

CI = confidence interval; NNT = number needed to treat.

**Figure 3:**
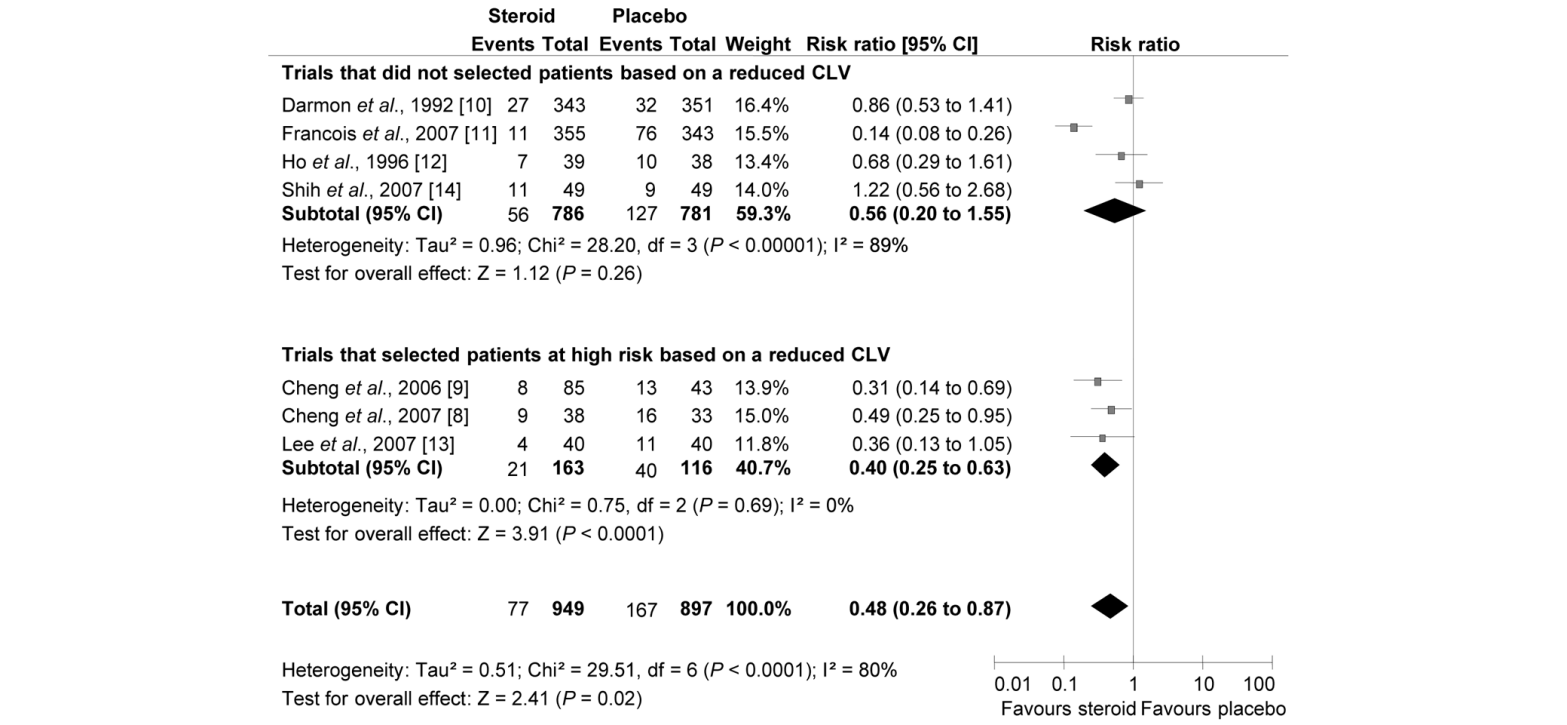
Risk ratio for post-extubation stridor according to the studied population. Risk ratios of post-extubation stridor rate for the individual randomised controlled trials comparing steroids with control groups and the pooled analysis. Vertical line = 'no difference' point between the two groups; squares = risk ratios (the size of each square denotes the proportion of information given by each trial); diamonds = pooled odds ratios for randomised controlled trials that did not select patients at high risk (upper) and trials that did selected patients at high risk, based on a reduced cuff leak volume (CLV; lower); horizontal lines = 95% confidence intervals (CI).

Stridor was described in the seven RCTs (Figure 3). Among the 897 patients who did not receive steroid therapy before extubation, 167 experienced symptomatic post-extubation stridor (18.6%; range = 9.1% to 48.5%; Figure 3). In one trial [13], 9 of 11 patients had severe respiratory distress that required non-invasive positive pressure ventilation. Of the 949 patients who received corticosteroids, 77 (8.1%; range = 2.8% to 23.7%) experienced symptomatic laryngeal obstruction (RR = 0.48, 95% CI = 0.26 to 0.87, *P *= 0.02; Figure 3). Eleven patients needed to be treated to prevent one patient from developing stridor (95% CI = 8 to 42) in the overall population (selected and unselected patients; Table 2). Aerosol with adrenaline (n = 19) and non-invasive positive pressure ventilation (n = 3) were used to treat laryngeal dyspnoea in the steroid group [9,12,13].

**Figure 4:**
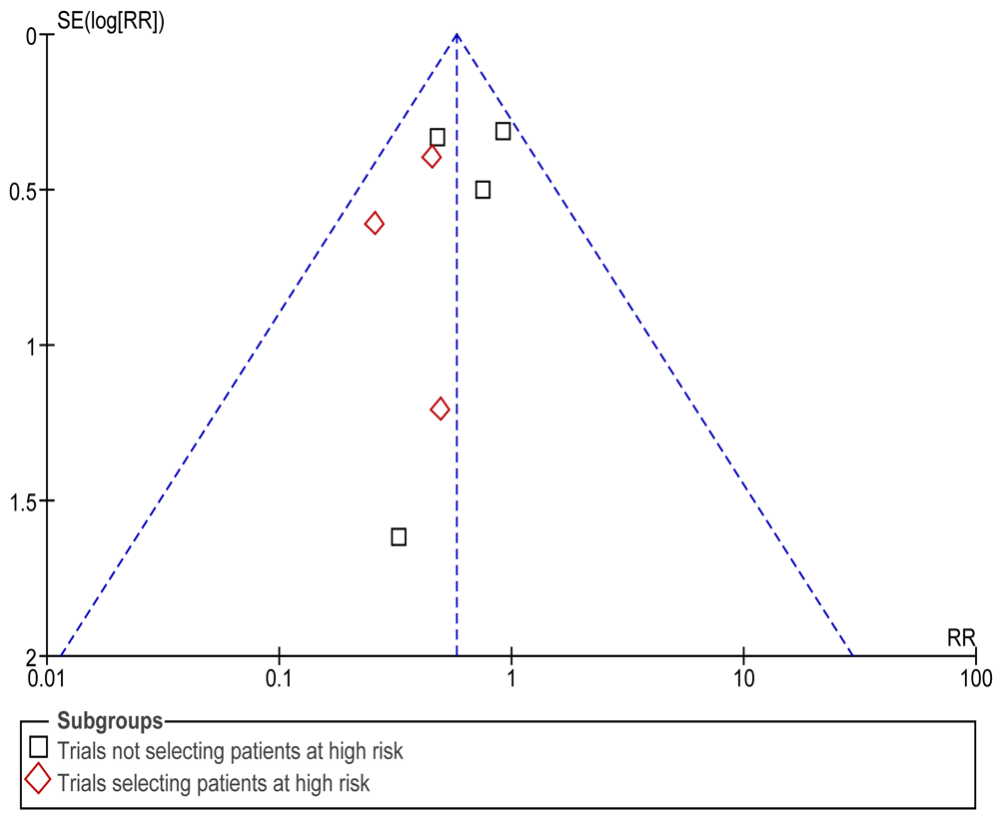
Funnel plot for outcome reintubation to detect bias or systematic heterogeneity in trials according to the studied population (selected vs unselected patients at risk based on a reduced cuff-leak volume). Each point represents one trial. SE = Standard Error. RR = Relative Risk.

Similar to reintubation, subgroup analysis was performed to evaluate patients at a higher risk for laryngeal dyspnoea. In high-risk patients, based on reduced cuff-leak volume, the overall incidence was 34.5% for the control groups and 12.9% in the steroid groups. In this context, the relative benefit was 0.40 (95% CI = 0.25 to 0.63, *P *< 0.001; NNT = 5, 95% CI = 4 to 8; Table 2). In contrast, steroids did not significantly reduce the incidence of post-extubation stridor when high-risk patients were not selected (RR = 0.56, 95% CI = 0.20 to 1.55; Figure 3). Moreover, the coefficient of heterogeneity (I^2^) was high, presumably explained by the trial performed by Francois and colleagues [11]. After exclusion of this study, the coefficient of heterogeneity was 0 (RR = 0.89, 95% CI = 0.61 to 1.30). A funnel plot of the treatment effect (logarithm RR of reintubation) versus trial precision was symmetric and centred around an RR of less than 1.0, suggesting that there is no publication bias or other biases (Figure 4).

No additional information with respect to outcomes of patients (death, duration of ventilation, infection and cost) that required reintubation was provided by the authors in the articles. Francois and colleagues [11] reported one death in each group; the reason was respiratory failure and septic shock in the placebo and corticosteroid groups, respectively. Five trials found that women have a significantly higher risk of symptomatic laryngeal oedema after extubation [9-12,14].

#### Outcomes according to when steroid administration was initiated before extubation: 'late' defined by starting less than two hours before planned extubation versus 'early' administration defined by starting steroid administration at least four hours (range = 4 to 24 hours) before planned extubation

**Figure 5:**
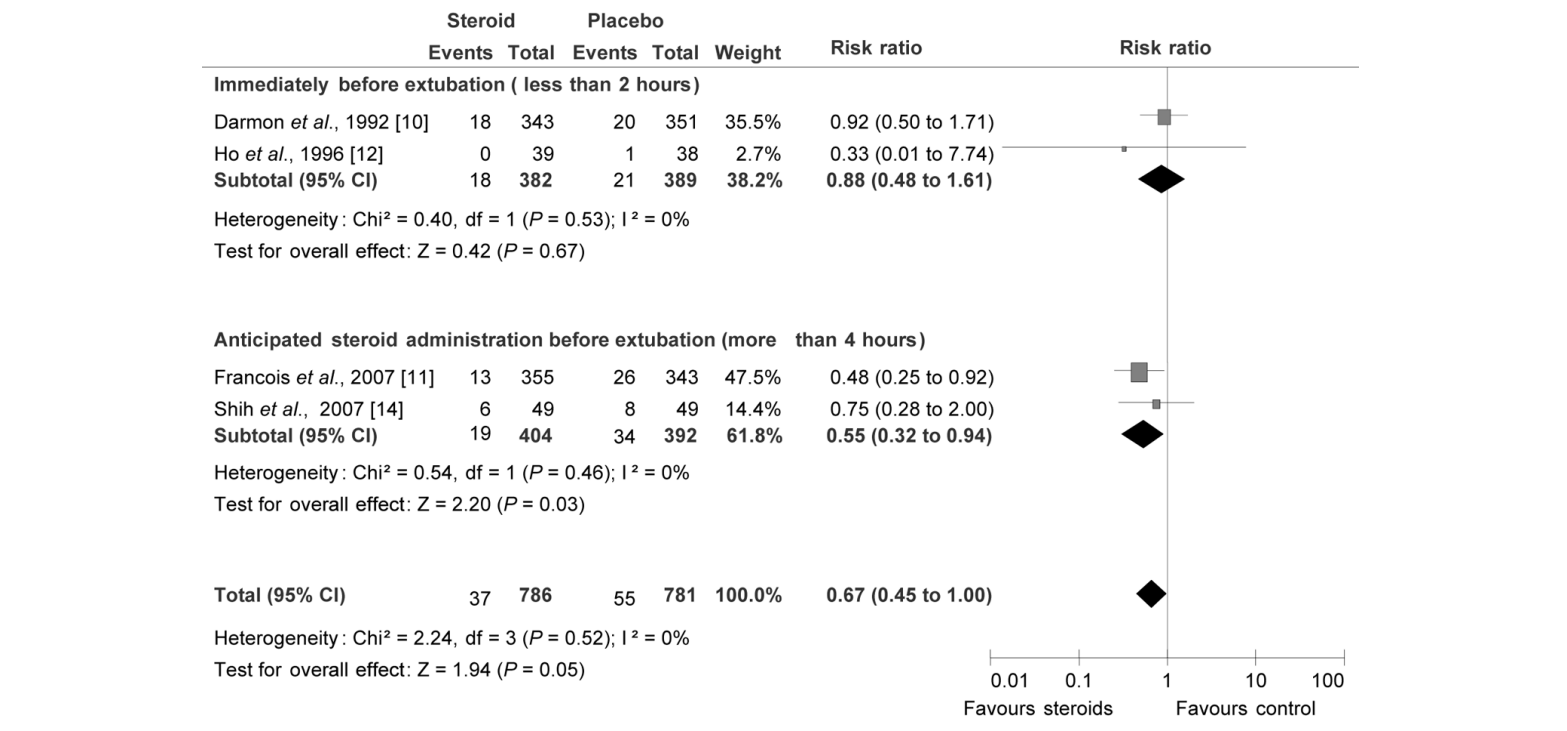
Risk for reintubation according to the steroid administration initiation timing before extubation in unselected patients. Risk ratios of reintubation rate for the individual randomised controlled trials comparing steroids with control groups and the pooled analysis. Vertical line = 'no difference' point between the two groups; squares = odds ratios (the size of each square denotes the proportion of information given by each trial); diamonds = pooled odds ratios for randomised controlled trials with for which steroid administration was started less than two hours before planned extubation (upper) and trials for which steroid administration was started at least four hours (ranged 4 to 24 hours) before planned extubation (lower); horizontal lines = 95% confidence intervals (CI). CLV = cuff-leak volume.

**Figure 6:**
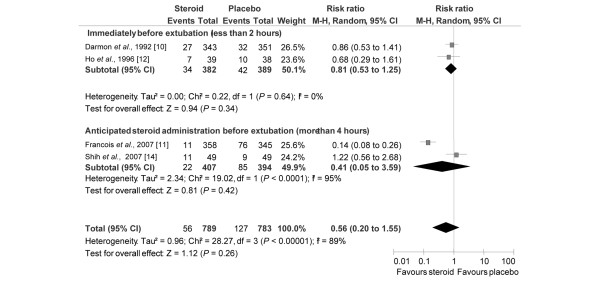
Risk for post-extubation stridor according to the timing steroid administration initiation before extubation in unselected patients. Risk ratios of post-extubation stridor rate for the individual randomised controlled trials comparing steroids with control groups and the pooled analysis. Vertical line = 'no difference' point between the two groups; squares = odds ratios (the size of each square denotes the proportion of information given by each trial); diamonds = pooled odds ratios for randomized controlled trials for which steroid administration was started less than two hours before planned extubation (upper) and trials for which steroid administration was started at least four hours (ranged 4 to 24 hours) before planned extubation (lower); horizontal lines = 95% confidence intervals (CI). CLV = cuff-leak volume.

In the subgroup of patients with a high risk for post-extubation stridor, steroids were always administrated early (more than four hours before the planned extubation; Figure 2). In contrast, timing of initiation of steroid administration varied from one trial to another when authors did not select patients at high risk. Among the four studies that included patients not selected as being at high risk [10-12,14], two trials used a protocol with an early injection, namely more than four hours before extubation [11,14], and the two others injected steroids just before the extubation [10,12]. Pooled together, these two trials [10,12] did not show that steroids decrease the risk of reintubation (RR = 0.88, 95% CI = 0.48 to 1.61; Figure 5) or stridor (RR = 0.81, 95% CI= 0.53 to 1.25; Figure 6). However, an anticipated administration of steroids (more than four hours before planned extubation) significantly decreases the risk of reintubation (RR = 0.55, 95% CI = 0.32 to 0.94; NNT = 26, 95% CI= 17 to 193; Figure 5) but not for stridor (RR = 0.41, 95% CI = 0.05 to 3.59; Figure 6).

## Discussion

The present meta-analysis documents that steroid administration before a planned extubation decreases the risk of post-extubation stridor and reintubation both in high-risk and unselected patients. The beneficial effect of steroids to prevent post-extubation stridor and reintubation was clear in the subgroup of patients at high-risk for development of post-extubation stridor as identified by a cuff-leak test (a low level of leak less than 110 ml or less than 25%).

The discrepancies observed in studies that evaluated the interest to administer steroids before extubation could be due to several factors including patient inclusion criteria, duration of intubation, dosage, timing of treatment and risk levels of developing stridor. Only the last two criteria (risk levels of developing stridor and timing of administration initiation) could be extensively evaluated in the present meta-analysis, allowing their importance to be reported for the first time. Post-extubation stridor is commonly the result of oedema of the subglottic area or the vocal cords. The difficulty in defining the relationship between laryngo-tracheal injury and post-extubation stridor is that the presence of the endotracheal tube precludes direct visualisation of the upper airway before extubation.

The ability to predict which patients will develop stridor following extubation, possibly culminating in reintubation, is obviously a desirable goal. Beyond assessment of risk factors, clinicians have long used the cuff-leak test to predict post-extubation airway patency, wherein the endotracheal tube cuff is deflated and a leak of air around the tube is sought during either spontaneous ventilation (with the endotracheal tube lumen occluded) or positive-pressure ventilation. The cuff-leak test may be performed using the 'qualitative method' (presence or absence of air leak around the tube when the cuff is deflated) or the 'quantitative method' by reporting the leak volume (inspired minus exhaled tidal volume during positive-pressure ventilation when the cuff is deflated) or the fraction of leak volume (inspired minus exhaled volume divided by inspired tidal volume when the cuff is deflated). Several cuff-leak test studies [5,9,27-30] suggest that the presence of an air leak is associated with a low likelihood of clinically important post-extubation stridor, whereas the absence or a low level of leak (less than 110 to 140 ml in absolute value or less than 12% to 25% in relative value) is associated with a high incidence of stridor and reintubation. The use of the cuff-leak test should be standardised and take into account a possible discrepancy between inspired and exhaled tidal volume measurement devices together with significant breath by breath variability.

A more reliable identification of patients at high-risk of developing post-extubation stridor and reintubation would appear desirable not only to decrease the risk of reintubation, but also to avoid excessive steroid treatment as it may induce adverse effects in patients for whom there is no need. Indeed as shown in the present meta-analysis, the NNT to prevent one stridor episode decreased from 11 in the overall population (selected and unselected) to five in a population determined to be at high-risk of developing post-extubation stridor as determined by the cuff-leak test (Figure 3 and Table 2). However, steroids did not significantly reduce the incidence of post-extubation stridor when patients were not selected (that is, unselected patients) for their risk of post-extubation stridor. The NNT to avoid one reintubation decreased from 28 in the overall population (selected and unselected) to nine in patients at high risk (Figure 2 and Table 2). On the other hand, the benefit of steroids is unclear when trials did not use the cuff-leak test to selected patients. In this case, the NNT increased to 44 and the upper limit of the CI is infinity (Figure 2 and Table 2).

Although steroids are potentially associated with several adverse effects (such as hyperglycaemia, arterial hypertension, agitation and infection) when they are administered for a few days (more than 48 hours) [31], side effects associated with steroid treatment for less than 24 hours are minimal [17,18]. The studies included in the present meta-analysis reported no side effects related to steroids, but detection of steroid-related adverse events was not specifically studied in these trials.

Laryngotracheal injury related to intubation may cause narrowing of the airway mainly due to inflammatory oedema. The potential capacity of steroids to relieve laryngeal oedema is mainly due to its anti-inflammatory effects, which inhibit the release of inflammatory mediators and decrease capillary permeability [9,11,13,18]. The initial anti-inflammatory effects start at least one to two hours after intravenous administration and maximal effects appear between 2 and 24 hours, depending on steroid type and administered dose [9,11,17,18]. Indeed, a single injection of dexamethasone (1 mg/kg) one hour before extubation had no effect on subglottic histological injury in a rabbit model [32,33]. Moreover, in the two trials [10,12] included in the present meta-analysis in which steroids were administered one hour before extubation, no significant difference was observed between control and steroid groups for post-extubation stridor and reintubation rates. The same is true for the study by Gaussorgues and colleagues [25] for which steroids were also administered one hour before extubation and no significant difference was observed between control and steroid groups for post-extubation stridor and reintubation rates. Although the study by Gaussorgues and colleagues [25] was excluded because the quality assessment score was less than three, the inclusion of this study [25] would not change the conclusions of the present meta-analysis. Except for one trial presented in abstract form at a congress [14], all the published RCTs in which steroids were administered at least 4 to 24 hours before extubation (Table 1 and Figures 5 and 6) reported a significant decrease in post-extubation stridor [8,9,11,13] and reintubation [8,9,11].

It might be argued that the use of corticosteroids in adult critical care for planned extubation is unnecessary, because objectively the incidence of reintubation is low and symptomatic laryngeal oedema has self-limited symptoms. However, stridor and laryngeal dyspnoea increase care needs because of the administration of adrenaline or corticosteroid aerosol and associated nursing time. Similarly, reintubation increases cost, morbidity, care needs, and both ICU and hospital lengths of stay. Unfortunately, trials included in the current meta-analysis evaluated the benefit of corticosteroids only during the first 48 hours and no information on the outcome of reintubated patients was provided. Further studies on this topic are needed; using standard criteria for the assessment of readiness to extubate and a well-defined evaluation on the relation between post-extubation laryngeal oedema and re-intubation.

The quality of the trials included in a systematic review may alter the results [34], because meta-analyses are often handicapped by the heterogeneity of the included trials. Moher and colleagues [34] demonstrated that meta-analyses with low-quality trials (Jadad assessment scale of two or less) compared with high-quality trials (Jadad assessment scale above two) were associated with a 33% increase in the estimated benefit. Similarly, trials using inadequate allocation concealment may also overestimate the benefit of treatment by as much as 37% [34]. Therefore, multiple scales have been proposed to assess the quality of trials included in a meta-analysis in order to decrease bias due to the inclusion of low-quality trials. We used the Jadad composite scale [20] to assess quality, using the following items: randomisation, double-blinding and patient withdrawals.

Meta-analyses of trials with low quality, as evaluated with this scale, significantly exaggerate benefits [19,34]. All seven trials selected for our systematic review have a scale reflecting high quality [34] and, consequently, were double-blinded and randomised. Patients included in trials have variable risks for post-extubation stridor or reintubation. Interestingly, the reduction of risk for stridor appears to be similar (approximately 50%), regardless of the risk of post-extubation laryngeal dyspnoea, suggesting that the effect is the same in the presence of oedema. Dosage, duration and type of corticosteroids differed from one trial to another. Pooling RCTs with varying designs may be interesting because the current meta-analysis appears to demonstrate that the timing of the first administration influences the risk of reintubation.

The current meta-analysis suggests an effect of administration timing on the efficacy of corticosteroids, because steroids appear to prevent reintubation more effectively if they are administrated at least four hours before planned extubation. As stridor and reintubation, secondary to upper obstruction airway obstruction, occur soon after extubation [5,11], it may be reasonable to suggest starting steroid treatment at least four hours before planned extubation to prevent prolongation of weaning from mechanical ventilation.

Further studies should be conducted to better define the optimal use of steroids to prevent extubation failure. In patients selected at high risk for postextubation stridor (for example, traumatic intubation, low cuff-leak value or previous extubation failure) steroids should be used but the optimal steroid to use before extubation without delay remains to be established, as does steroid type, dosing regimen, administration timing and duration. Dose response should also be established to achieve the lowest effective dose. Moreover, the risk of steroid use remains a source of concern in critical care patients. The side effects of steroid administration to prevent reintubation are unknown and were not investigated clearly in all trials included in this meta-analysis. The current meta-analysis showed no benefit when trials that did not select patients at risk for reintubation were pooled. In this group, only one trial [5,11] found a significant benefit of steroid use but the others found no benefit. The study by Francois and colleagues [11] appears to be the main cause of heterogeneity between the trials that did not select patients at risk. The timing of administration does not seem to be the major reason for heterogeneity because the study by Shih and colleagues [14] administrated steroid sooner than Francois and colleagues [11] (24 hours compared with 12 hours, respectively). Another hypothesis may be the dose of steroid used by Francois and colleagues [11] because they administrated the highest dose among all trials studied. Finally, all trials have the possibility of giving a significant result even if one is not available (Type I error). Thus, the evidence for steroid administrated in unselected patients remains unclear and additional studies are warranted to clearly determine the benefits, but also the potential adverse effects, of this group of drugs.

## Conclusions

The present meta-analysis suggests a beneficial effect of steroids to prevent post-extubation stridor and reintubation was observed in the subgroup of patients with a high risk of developing post-extubation stridor, as identified by the cuff-leak test, and that steroid treatment before a planned extubation decreases the risk of reintubation only if intravenous steroid administration was performed at least four hours before planned extubation. The benefit of steroids remains unclear when high-risk patients are not selected.

## Key messages

• A high-risk population to develop post-extubation stridor and reintubation can be identified by a cuff-leak test (a low level of leak less than 110 ml or less than 25%).

• There is convincing evidence for giving steroid therapy at least four hours before extubation to prevent stridor and reintubation in a high-risk population.

• The steroid benefit remains unclear when patients are not selected.

## Abbreviations

CI: confidence interval; ICU: intensive care unit; NNT: number needed to treat; RCT: randomised controlled trial; RR: relative risk.

## Competing interests

The authors declare that they have no competing interests.

## Authors' contributions

SJ designed and supervised the research, collected, analysed and interpreted the data, drafted and revised the manuscript. BJ contributed to the conception of the study and approved the final version of the manuscript. GC made substantial contributions to the conception and design of the study and approved the final version of the manuscript. FB participated in the design of the study and helped to draft the manuscript. EM co-designed and supervised the research, collected and analysed the data and performed the statistical analysis. All authors read and approved the final manuscript.
